# Supplementary figures and images for: Beta and Theta Oscillations Correlate With Subjective Time During Musical Improvisation in Ecological and Controlled Settings: A Single Subject Study
Source: Front Neurosci. 2021 Jun 10;15:626723. doi: 10.3389/fnins.2021.626723 (PMC8222590; doi:10.3389/fnins.2021.626723)

**a.**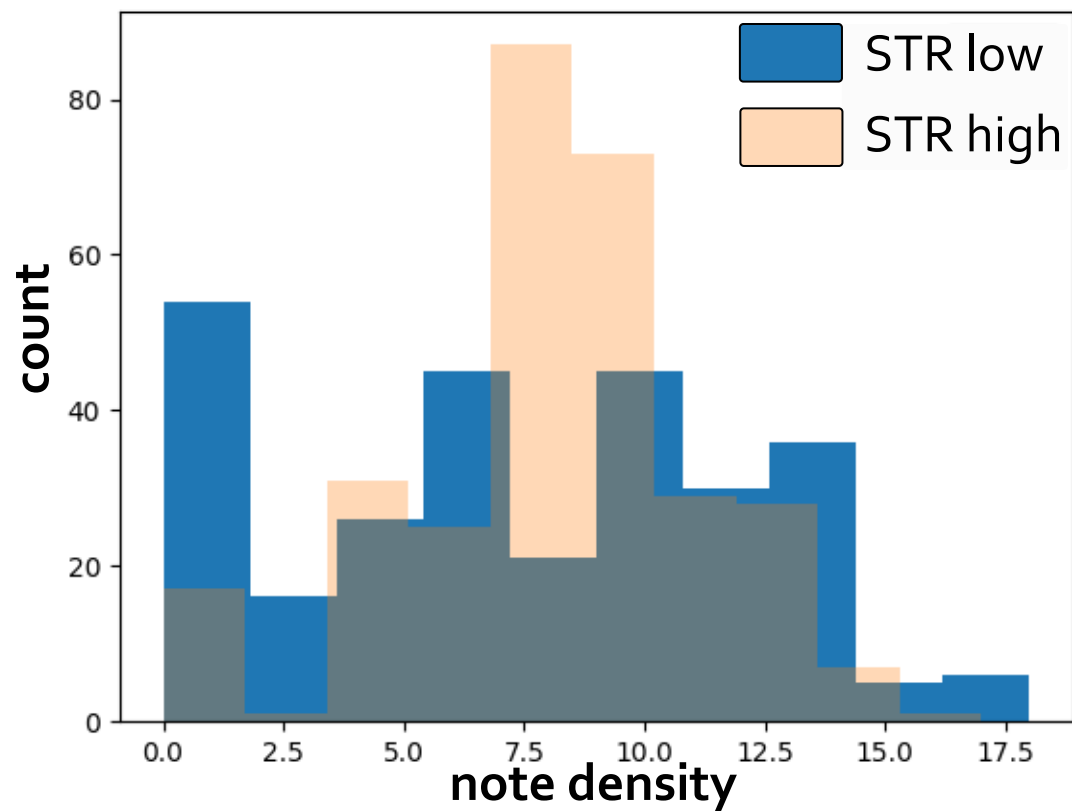**b.**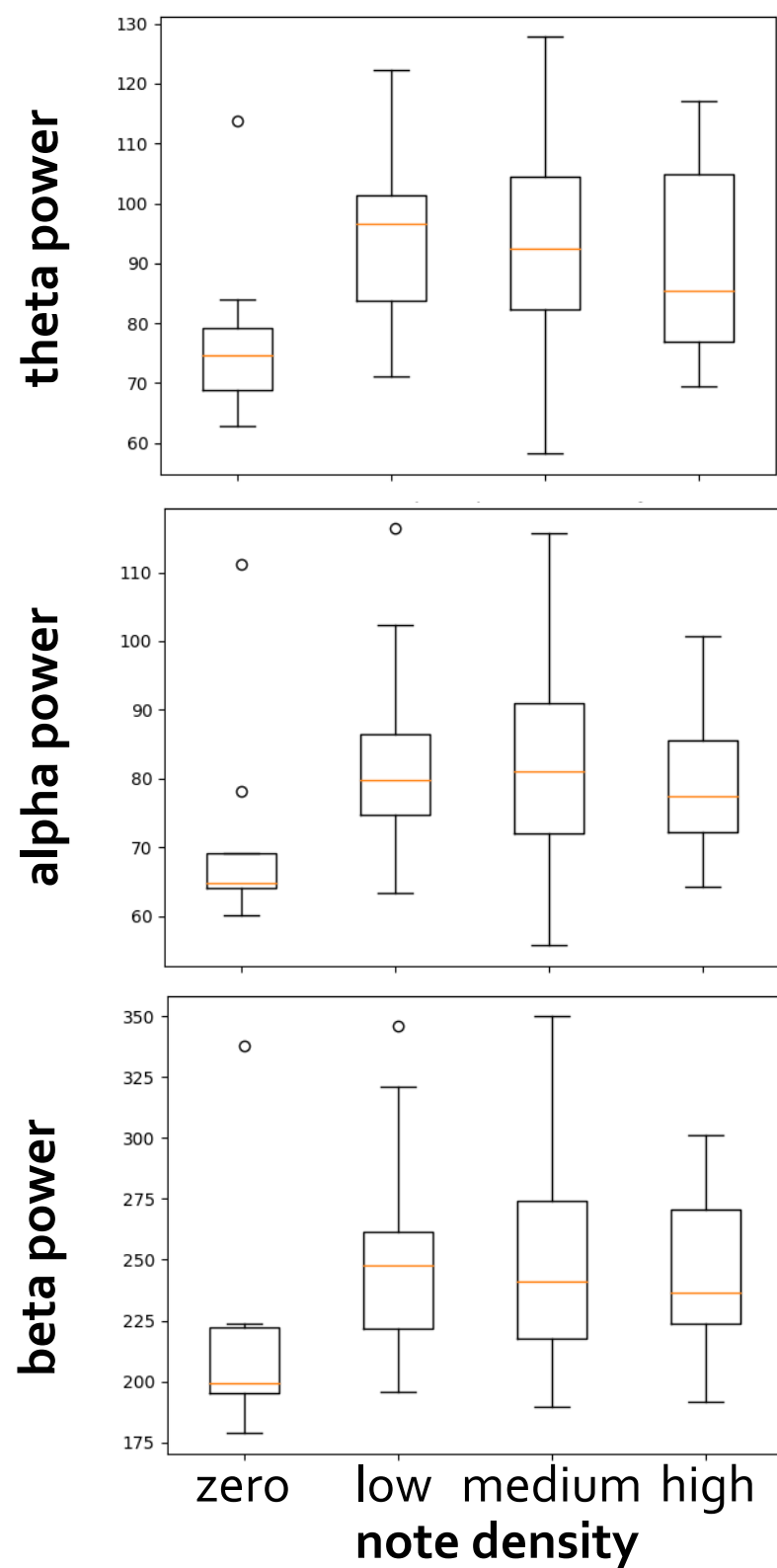

Supplement: Supplementary Figure 1 — Audio Analysis results for performance 1. (A) Shows note density histograms for STR low (blue) and high (orange). (B) Reports EEG power changes as a function of the note density in the three frequency bands of interest (theta, alpha, beta). [file Data_Sheet_1.PDF]

**a.**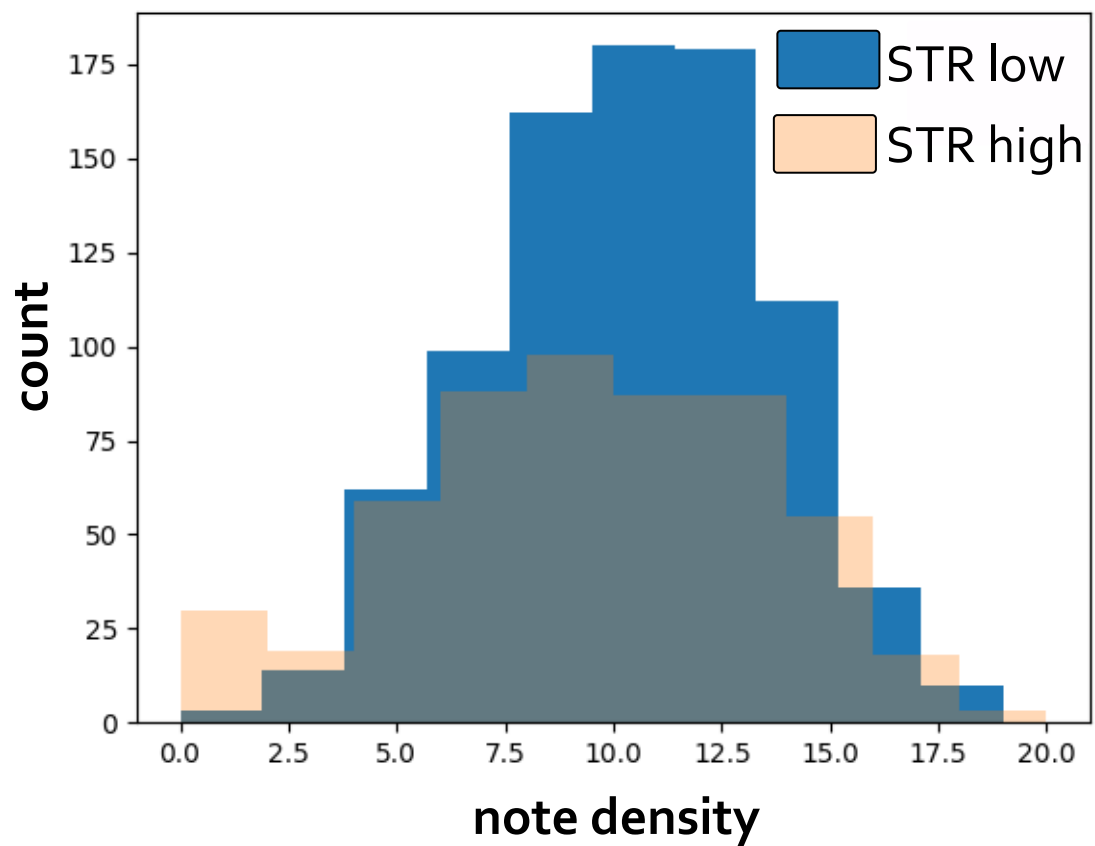**b.**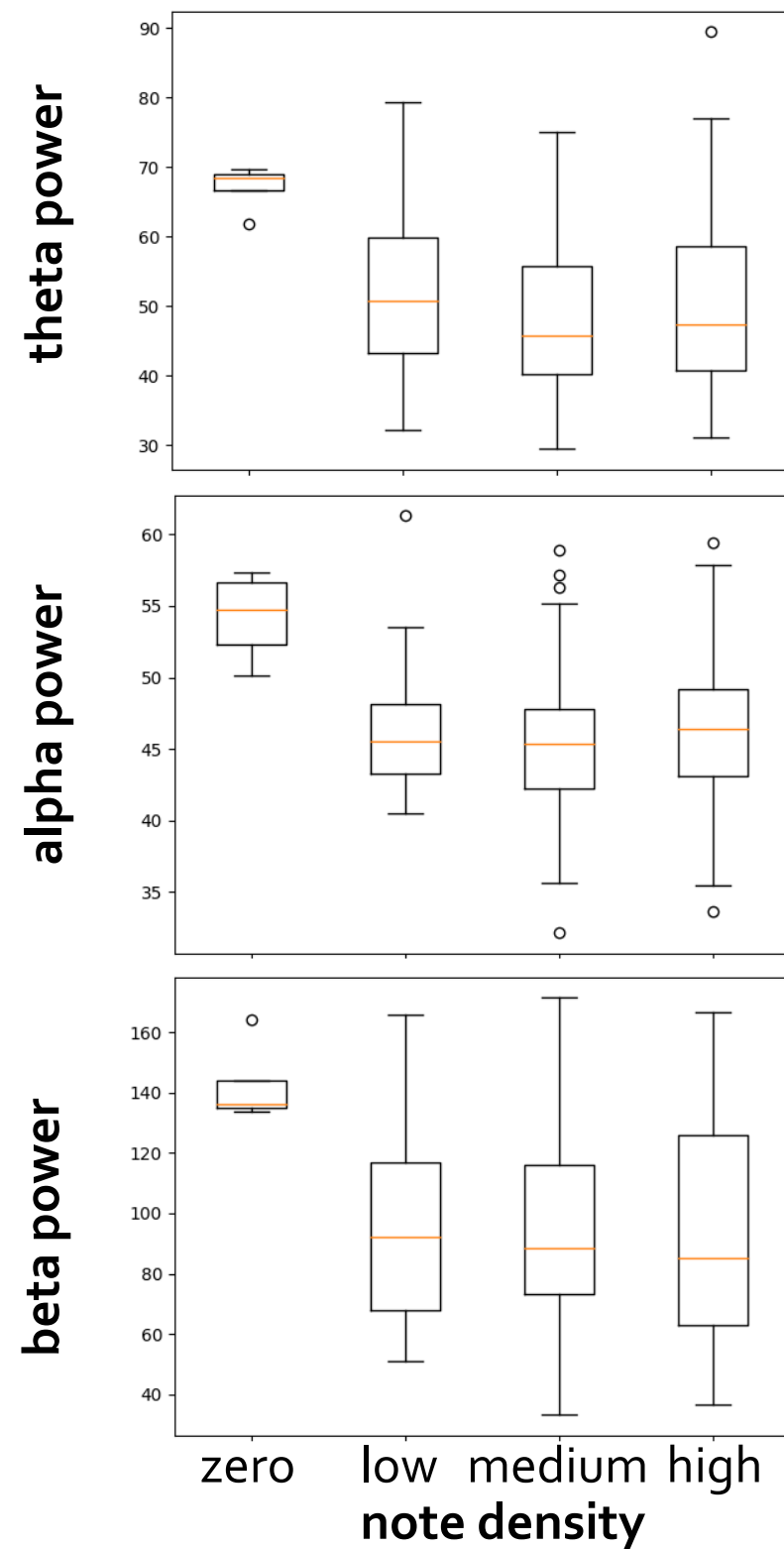

Supplement: Supplementary Figure 2 — Audio Analysis results for performance 3. (A) Shows note density histograms for STR low (blue) and high (orange). (B) Reports EEG power changes as a function of the note density in the three frequency bands of interest (theta, alpha, beta). [file Data_Sheet_2.PDF]
